# m^6^A-mediated ZNF750 repression facilitates nasopharyngeal carcinoma progression

**DOI:** 10.1038/s41419-018-1224-3

**Published:** 2018-12-05

**Authors:** Panpan Zhang, Qiuping He, Yuan Lei, Yingqin Li, Xin Wen, Mengzhi Hong, Jian Zhang, Xianyue Ren, Yaqin Wang, Xiaojing Yang, Qingmei He, Jun Ma, Na Liu

**Affiliations:** 1 State Key Laboratory of Oncology in South China, Collaborative Innovation Centre of Cancer Medicine, Guangdong Key Laboratory of Nasopharyngeal Carcinoma Diagnosis and Therapy, Sun Yat-sen University Cancer Center, 510060 Guangzhou, China; 2 Zhongshan School of Medicine, Sun Yat-sen University, 510080 Guangzhou, China; 3 Guangdong Provincial Key Laboratory of Stomatology, Guanghua School of Stomatology, Sun Yat-sen University, 510055 Guangzhou, Guangdong China

## Abstract

Nasopharyngeal carcinoma (NPC) progression is regulated by genetic, epigenetic, and epitranscript modulation. As one of the epitranscript modifications, the role of N6-Methyladenosine (m^6^A) has not been elucidated in NPC. In the present study, we found that the poorly methylated gene *ZNF750* (encoding zinc finger protein 750) was downregulated in NPC tumor tissues and cell lines. Ectopic expression of *ZNF750* blocked NPC growth in vitro and in vivo. Further studies revealed that m^6^A modifications maintained the low expression level of *ZNF750* in NPC. Chromatin immunoprecipitation sequencing identified that ZNF750 directly regulated *FGF14* (encoding fibroblast growth factor 14), ablation of which reversed ZNF750’s tumor repressor effect. Moreover, the ZNF750-FGF14 signaling axis inhibited NPC growth by promoting cell apoptosis. These findings uncovered the critical role of m^6^A in NPC, and stressed the regulatory function of the ZNF750-FGF14 signaling axis in modulating NPC progression, which provides theoretical guidance for the clinical treatment of NPC.

## Introduction

Nasopharyngeal carcinoma (NPC) is a malignant head and neck cancer with apparent regional aggregation^1–3^. With the advancement of intensity-modulated radiation therapy and combined chemotherapy, great progress has been made in local and regional control of NPC. However, there are still about 30% of patients with NPC develop distant metastasis and/or recurrence^4^. Revealing the underlying mechanism governing NPC progression would identify novel targets to develop clinical treatment strategies.

Our previous genome-wide methylation profiling study revealed the methylation status between 24 NPC tissues and 24 normal nasopharyngeal epithelial tissues, from which a list of hypermethylated and hypomethylated genes was composed (GSE52068)^5^. Zinc Finger Protein 750 (ZNF750), as a transcription factor belonging to one of the Zinc Finger Protein family members, was the top-ranked hypomethylated gene in the dataset. Previous findings revealed that ZNF750 serves as a tumor repressor in oral squamous cell carcinoma^6^ and esophageal squamous cell carcinoma^7^. However, the mechanism by ZNF750 governs tumorigenesis and the role of ZNF750 in NPC remain largely unknown.

N^6^-methyladenosine (m^6^A) is the most common mRNA internal modification in eukaryotic organisms^8–10^. m^6^A mRNA methylation is catalyzed by multicomponent methyltransferases, among which methyltransferase like 3 (METTL3) and METTL14 have been characterized^11,12^. The methylated mRNA is recognized by protein “readers” YTH N6-methyladenosine RNA binding protein 1–3 (YTHDF1–3)^9,13^, which regulate mRNA stability and localization in the cell^14^. The importance of m^6^A modification in cancer progression is only beginning to emerge. Previous studies showed that AlkB homolog 5 (ALKBH5), as the RNA demethylase of m^6^A, mediates the promotion of breast cancer stem cell phenotype by elevating NANOG expression in the hypoxic environment^15^. Moreover, in acute myeloid leukemia (AML) cells, METTL3 was abundantly expressed and promoted *MYC*, *BCL2*, and *PTEN* translation through m^6^A modification, which inhibited cell differentiation and fueled leukemia progression^16^. However, the possible function of m^6^A in NPC is still completely unknown.

In this study, we identified that *ZNF750*, as a hypomethylated gene, was downregulated in NPC tumor tissues and cell lines. Overexpression of *ZNF750* inhibited the growth of NPC cells in vitro and in vivo. An m^6^A RNA immunoprecipitation (RIP) assay revealed that m^6^A was enriched in the *ZNF750* coding sequence (CDS) and contributed to *ZNF750*’s low expression in NPC. Moreover, chromatin immunoprecipitation sequencing (ChIP-Seq) identified fibroblast growth factor 14 (FGF14) as the downstream target of ZNF750, and blocking FGF14 reversed the tumor repressor effect of ZNF750 in NPC cells. These findings revealed the essential regulatory role of the ZNF750-FGF14 signaling axis and highlighted the importance of m^6^A modification in modulating gene expression post-transcriptionally in NPC.

## Results

### *ZNF750* is downregulated in NPC biopsy samples and cell lines

**Fig. 1:**
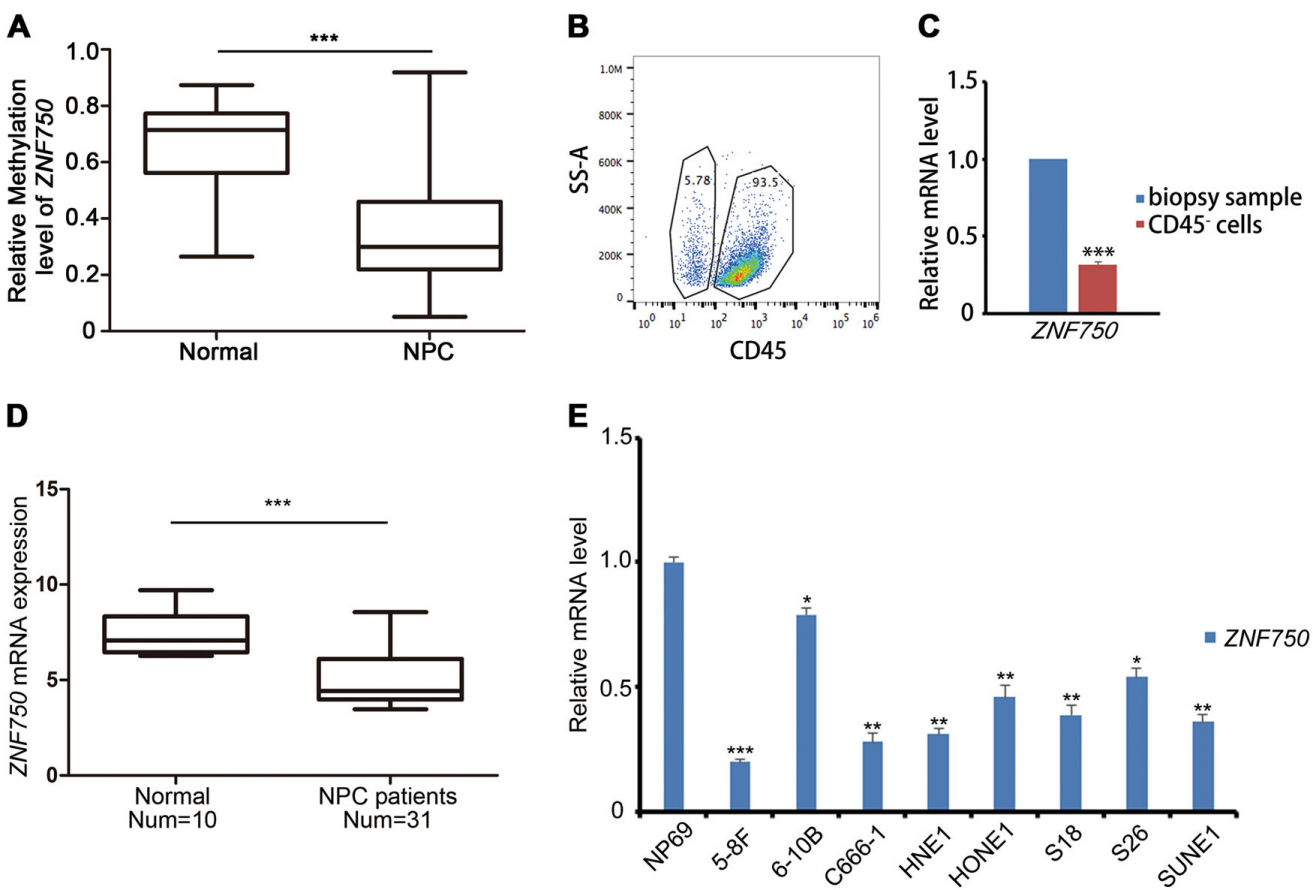
ZNF750 is downregulated in NPC biopsy samples and cell lines. **a** Relative methylation level of the *ZNF750* promoter region (5 kb upstream of transcription start site) in healthy controls (*n* = 24) and NPC patients (*n* = 24). **b** Flow cytometry sorting of CD45^−^ cells in NPC biopsy samples. **c** Quantitative RT-PCR detection of *ZNF750* expression in the whole biopsy samples and their matching CD45^−^ cells (*n* = 3). **d** ZNF750 expression in healthy controls and patients with NPC in the GEO dataset (GSE81687280). **e** Quantitative RT-PCR detection and comparison of *ZNF750* expression in normal nasopharyngeal epithelial cell line NP69 and various NPC cell lines. *p < 0.05, **p < 0.01, ***p < 0.001

Despite previous findings indicating that *ZNF750* is frequently mutated in head and neck squamous cell carcinoma (HNSC)^17,18^ and esophageal carcinoma (ESCA)^19^, *ZNF750* was not mutated in the majority of HNSC patients in the cBioPortal dataset^20,21^ (Figure S1A, B). In our previous NPC methylation dataset (GSE52068), *ZNF750* was identified as hypomethylated (Fig. 1a). However, the mRNA expression level of *ZNF750* did not seem to be correlated with its methylation status in HNSC (Figure S1B). To identify the expression level of *ZNF750* in NPC tissue samples, CD45^−^ cells were sorted to avoid the contamination from lymphocyte cells (Fig. 1b). *ZNF750* expression was decreased in CD45^−^ cells in NPC samples (*n* = 25) (Fig. 1c). In The Cancer Genome Atlas (TCGA) dataset, *ZNF750* expression was significantly downregulated in ESCA, HNSC, and skin cutaneous melanoma (SKCM) (Figure S1C). We then compared *ZNF750* mRNA expression levels between normal nasopharynx and NPC tissue samples using the Gene Expression Omnibus (GEO) dataset. Compared with that in normal tissues, the expression of *ZNF750* was significantly downregulated in NPC tissue samples (Fig. 1d). Moreover, in NPC cell lines, *ZNF750* expression was also significantly decreased (Fig. 1e). These results suggested that the expression of *ZNF750* was frequently downregulated in NPC, regardless of its methylation status.

### ZNF750 represses NPC growth in vitro and in vivo

**Fig. 2:**
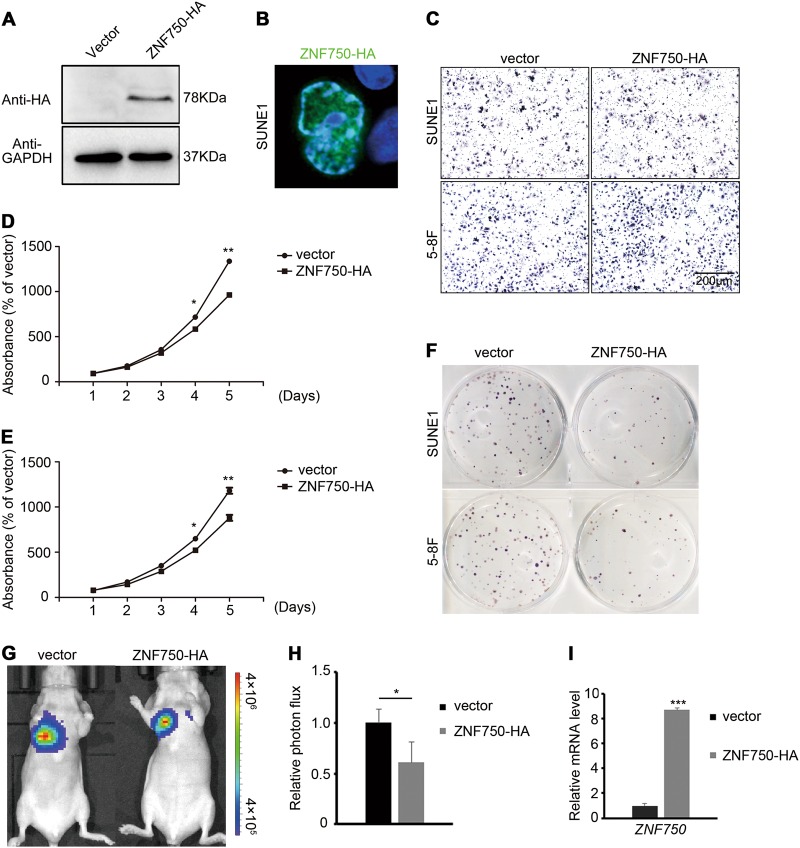
ZNF750 represses NPC growth in vitro and in vivo. **a** Western blotting detection of ZNF750-HA expression in SUNE1 cells. **b** Immunofluorescence detection of HA staining in SUNE1 cells with ZNF750-HA overexpression. **c** Transwell assay of SUNE1 and 5–8 F cells with or without ZNF750-HA ectopic expression. **d**, **e** CCK-8 assay of SUNE1 and 5–8 F cells with or without ZNF750-HA ectopic expression. **f** Colony formation assay of NPC cells with and without ZNF750-HA ectopic expression. **g**, **h** Tumor cell absorbance intensity and the quantification analysis of vector (*n* = 5) and *ZNF750* overexpressed (*n* = 6) groups 2 weeks after tumor cell implantation in mice. **i** The mRNA expression level of *ZNF750* in xenograft tumors. *p < 0.05, **p < 0.01

To study the function of ZNF750 in NPC, we firstly overexpressed *ZNF750* in NPC cells (Fig. 2a). We observed that ZNF750 was localized in cell nucleus (Fig. 2b), indicative of its transcriptional regulatory role in NPC cells. We then detected the NPC cell invasive and proliferative abilities of SUNE1 and 5–8 F that overexpressed *ZNF750*. The results showed that the invasive ability of NPC cells were not affected, while cell growth and colony formation was reduced significantly (Fig. 2c–f). To test the tumor repressor role of ZNF750 in vivo, SUNE1 cells with luciferase activity were subcutaneously injected into nude mice. We found that ectopic expression of *ZNF750* blocked tumor cell growth (Fig. 2g–i). Moreover, the HNSC patients with higher expression levels of ZNF750 displayed better disease-free survival (DFS) compared with those with low ZNF750 levels (Figure S1D). These results revealed that ZNF750 acts as a tumor repressor in NPC.

### m^6^A maintains the mRNA stability of ZNF750 in NPC cells

**Fig. 3:**
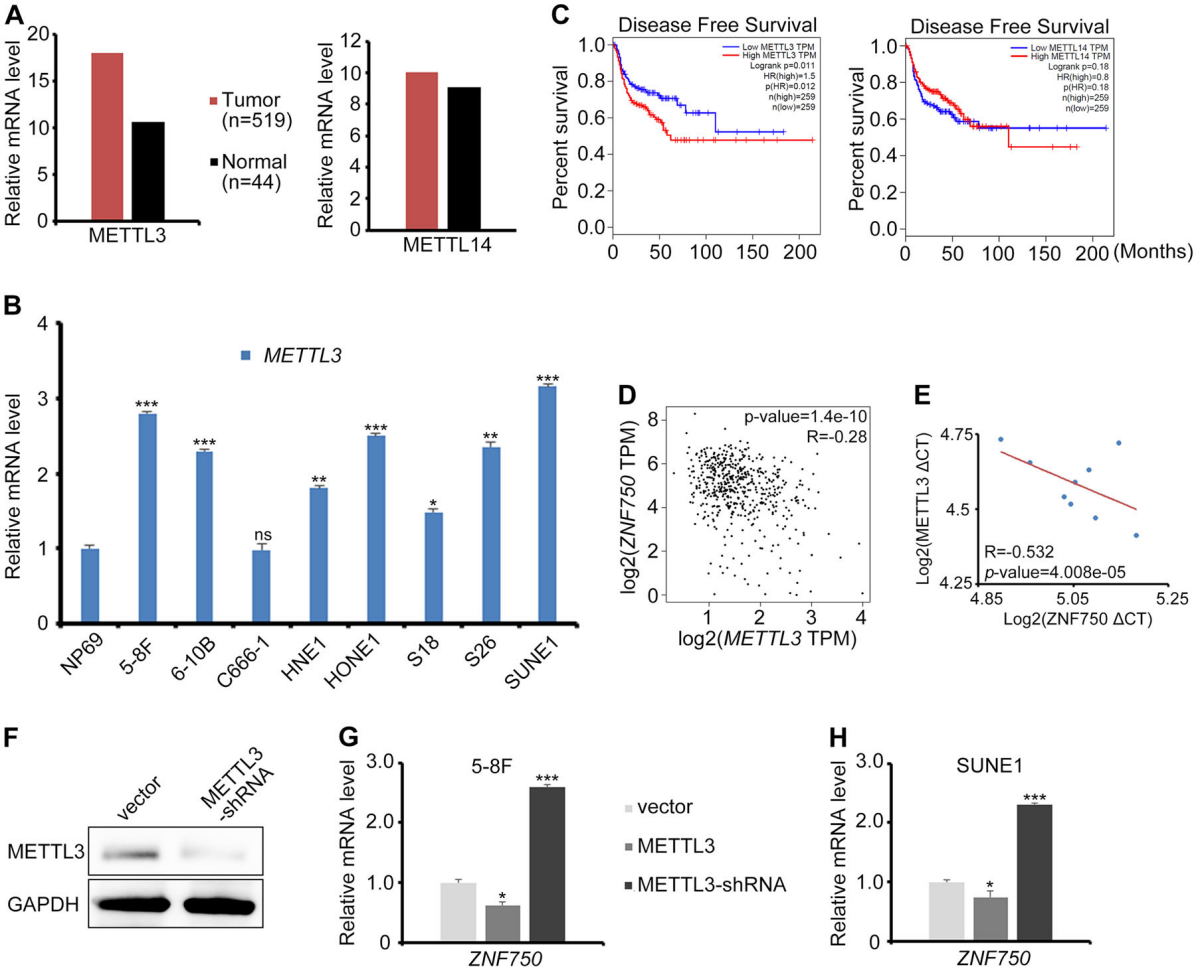
METTL3 functions as an oncogene in NPC. **a** *METTL3* and *METTL14* mRNA expression level in HNSC in the TCGA dataset. **b** The relative mRNA expression of *METTL3* in normal nasopharyngeal epithelial cell NP69 and various NPC cell lines, as assessed by quantitative RT-PCR. **c** Disease-free survival (DFS) analysis in HNSC according to the METTL3 or METTL14 expression in the TCGA dataset. **d** The correlation of *METTL3* and *ZNF750* mRNA expression in HNSC, as assessed by Pearson analysis. **e** Pearson analysis of the correlation between ZNF750 and METTL3 mRNA expression in NPC cell lines. The blue dots represent ZNF750 and METTL3 mRNA expression in NP69, 5–8 F, 6–10B, C666–1, HNE1, HONE1, S18, S26, and SUNE1 cells. CT: cycle threshold. **f** Western blotting detection of METTL3 levels in NPC cells with shRNA knock down of METTL3. **g**, **h** Quantitative RT-PCR detection of *ZNF750* expression in NPC cells with METTL3 ectopic expression or knockdown. *p *<* 0.05, **p *<* 0.01, ***p *<* 0.001

In our previous NPC methylation dataset (GSE52068), *ZNF750* was listed as the top hypomethylated gene in NPC patients (Supplementary Table 1). Considering the low mRNA expression level of ZNF750 in NPC, we wondered whether there is a post-transcriptional modification governing the mRNA stability of ZNF750. The methyltransferases METTTL3 or METTL14 were responsible for modulating mRNA stability post-transcriptionally. However, only METTL3 displayed an apparent increase in HNSC tissues and NPC cell lines (Fig. 3a, b), and the high expression level of METTL3 correlated with poor DFS in HNSC patients (Fig. 3c). Moreover, the METTL3 expression level was negatively correlated with the ZNF750 expression level in HNSC to some extent (Fig. 3d), and in NPC cell lines (Fig. 3e). Knocking down *METTL3* stimulated endogenous ZNF750 expression, and vice versa (Fig. 3f–h).

**Fig. 4:**
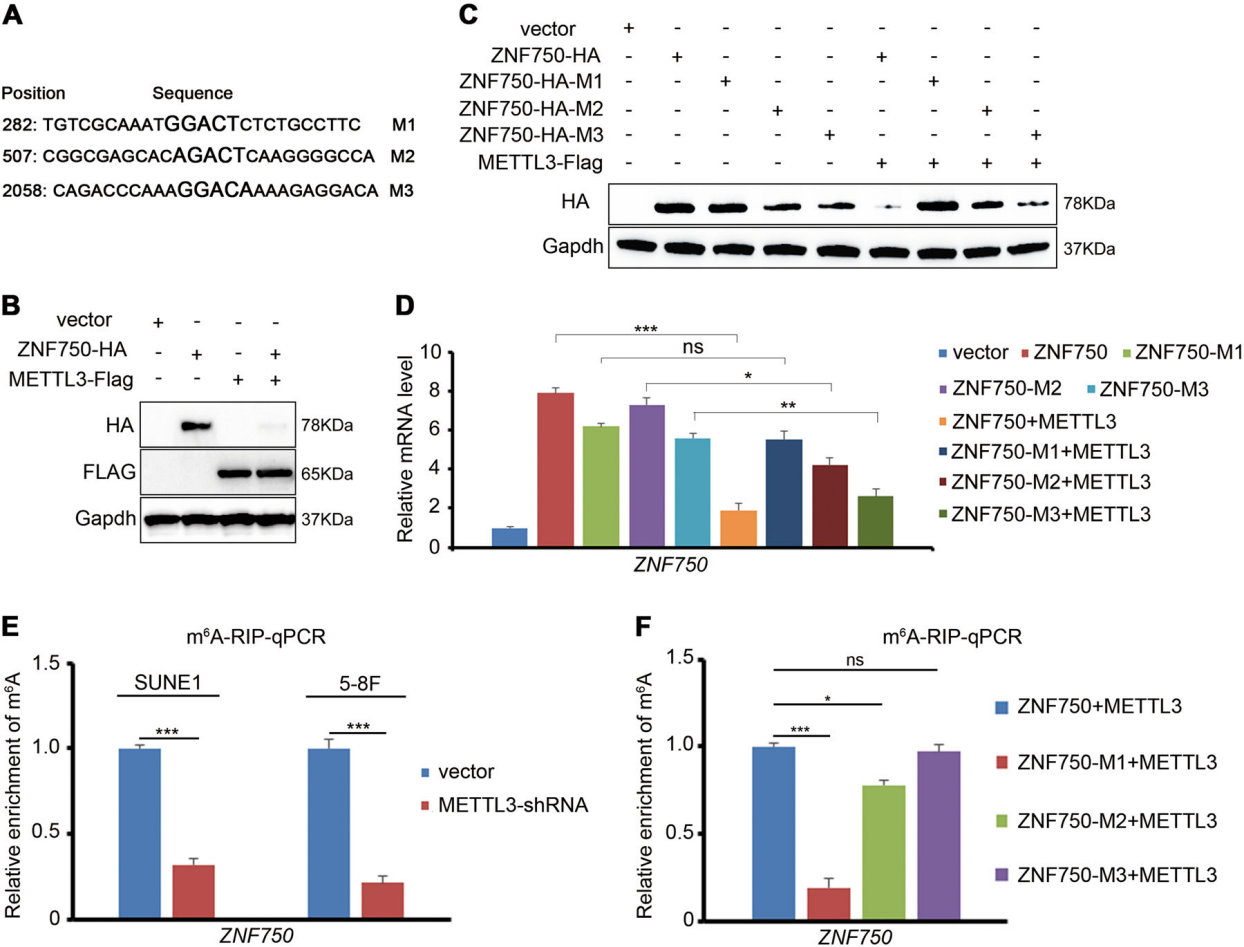
m^6^A represses ZNF750 expression in NPC cells. **a** Predicted m^6^A sites (the larger characters) in the coding sequence of *ZNF750*. **b** Western blotting detection of the ZNF750-HA expression after overexpressing METTL3. **c**, **d** The combined protein or mRNA expression of ZNF750-HA and its m^6^A sites mutated counterparts with or without METTL3-Flag overexpression. **e** The examination of m^6^A enrichment in endogenous *ZNF750* mRNA in NPC cells. m^6^A RIP assay was performed in the same amount of NPC cells with or without METTL3 knocking down, and the relative mRNA level of *ZNF750* was detected by quantitative RT-PCR. **f** The comparison of m^6^A enrichment in *ZNF750* mRNA and its mutated counterparts in NPC cells. m^6^A RIP assay was performed in the same amount of NPC cells with METTL3 overexpression, and the relative mRNA level of *ZNF750* was detected by quantitative RT-PCR. *p < 0.05, ***p < 0.001

The online tool SRAMP predicted three m^6^A recognition sites with high confidence in the coding sequence (CDS) of *ZNF750* (Fig. 4a). To confirm whether the m^6^A modification is applied to RNA after transcription, we overexpressed *METTL3* in NPC cells. The western blotting results showed that ZNF750 expression level was dramatically reduced (Fig. 4b). Then, the three m^6^A recognition sites in the *ZNF750* CDS region were mutated respectively (GAC to GCC). Upon *METTL3* overexpression, both the ZNF750-M2 and ZNF750-M3 displayed an apparent decrease (Fig. 4c, d), indicating that m^6^A modulated *ZNF750* expression mainly through the M1 region in NPC cells, despite the fact that M2 and M3 might also be recognized to some extent. To confirm the existence of the m^6^A modification in the *ZNF750* CDS directly, an m^6^A RIP assay in NPC cells was performed. Compared with its counterpart control, m^6^A enrichment in *ZNF750* was significantly decreased upon *METTL3* knockdown (Fig. 4e), supporting the view that *ZNF750* mRNA possessed *METTL3-*dependent m^6^A modifications. Moreover, consistent with the results shown in Fig. 3c, most of the m^6^A was found to be enriched in the M1 region of *ZNF750* (Fig. 4f). These results demonstrated that m^6^A maintained the mRNA stability of *ZNF750* in NPC cells.

### ZNF750 functions through modulating FGF14 expression

**Fig. 5:**
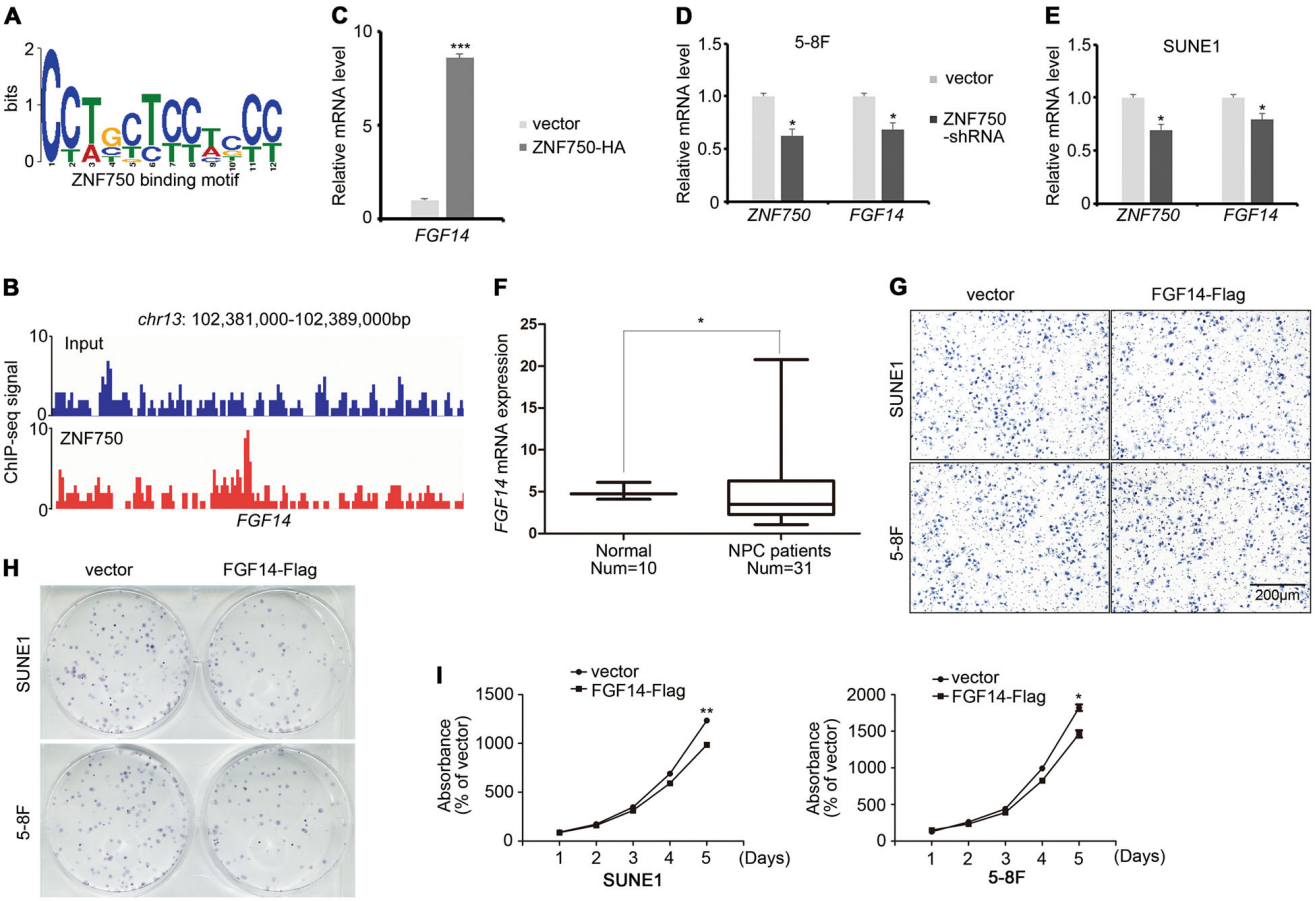
FGF14 functions as a tumor repressor in NPC. **a** Binding motif of ZNF750 identified by ChIP-Seq. **b** A screenshot of the ZNF750 ChIP-Seq signal at the *FGF14* locus. **c** *FGF14* expression in ZNF750 overexpressing SUNE1 cells, as assessed by quantitative RT-PCR. **d**, **e** The mRNA expression of *ZNF750* and *FGF14* in NPC cells with ZNF750 knockdown. **f** *FGF14* expression in healthy controls and patients with NPC in the GEO dataset (GSE81687280). **g** Transwell assay of NPC cells with or without FGF14 overexpression. **h** Colony formation assay of NPC cells with or without FGF14 overexpression. **i** CCK-8 assay of NPC cells with or without FGF14 overexpression. *p < 0.05, **p < 0.01, ***p < 0.001

We next addressed the functional mechanism of ZNF750 in repressing NPC growth. ChIP-Seq against *ZNF750* was performed to explore its downstream targets (Fig. 5a), among which FGF14 was found to be highly enriched (Fig. 5b, Supplementary Table 2). Quantitative RT-PCR confirmed that *FGF14* was upregulated upon ZNF750 overexpression (Fig. 5c), and was downregulated upon *ZNF750* knockdown (Fig. 5d, e). Moreover, the expression of *FGF14* was also decreased in patients with NPC (Fig. 5f). To identify the role of FGF14 in NPC, we detected the invasive and proliferative ability of NPC cells overexpressing *FGF14*. The results showed that FGF14 did not influence cell invasion (Fig. 5g). However, the growth and colony formation abilities of NPC cells were significantly reduced (Fig. 5h, i), supporting the view that FGF14 acts as a tumor repressor in NPC.

**Fig. 6:**
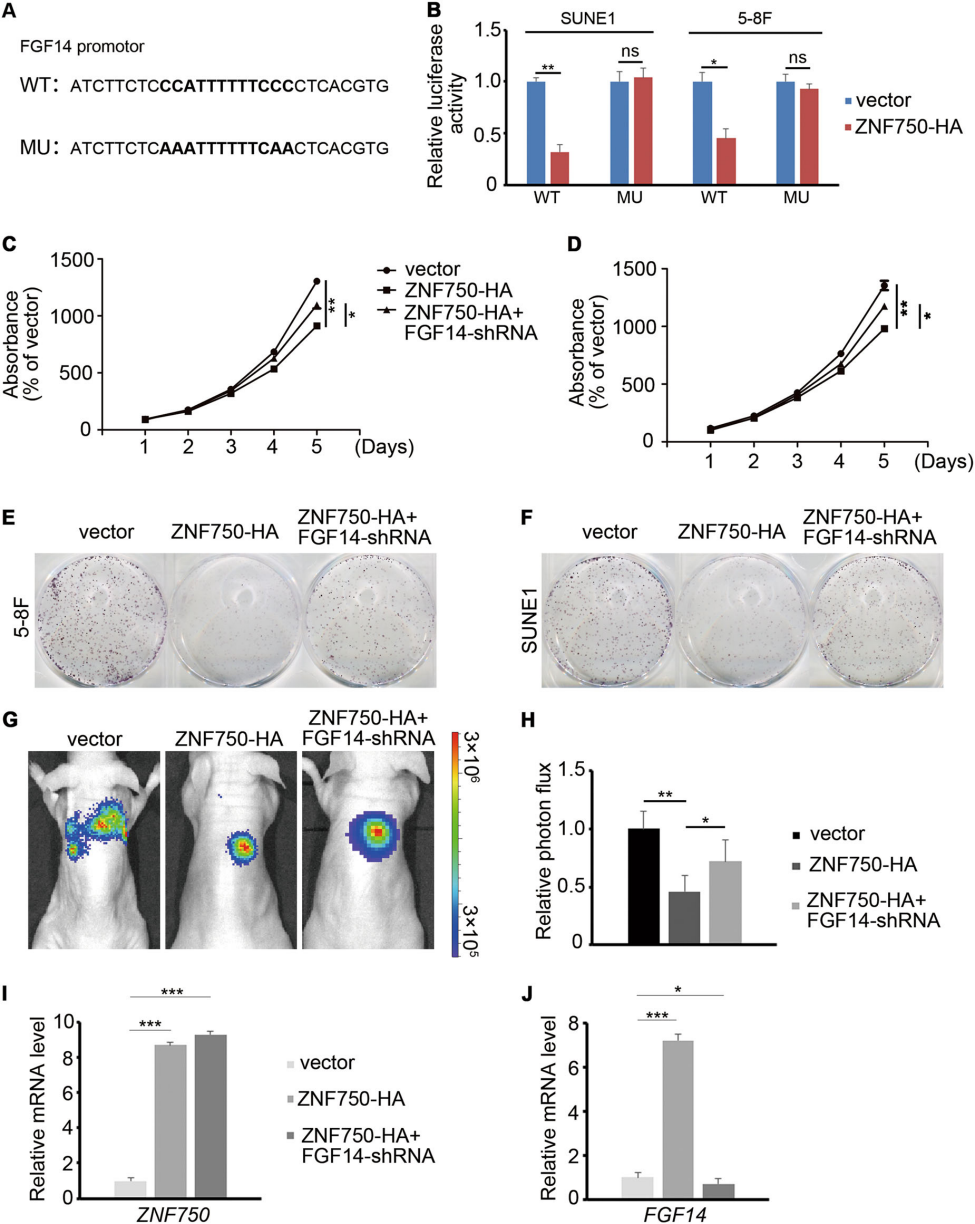
*FGF14* serves as the downstream target of ZNF750. **a** ZNF750 binding site with and without mutation in the promoter of *FGF14*. **b** Luciferase activity of FGF14 wild-type or mutated NPC cells with or without ZNF750 overexpression. **c**, **d** CCK-8 assay of NPC cells with ZNF750 overexpression or FGF14 knockdown. **e**, **f** Colony formation assay of NPC cells with ZNF750 overexpression or FGF14 knockdown. **g**, **h** Tumor cell absorbance intensity and the quantification analysis of vector (*n* = 5), ZNF750 overexpression (*n* = 5), and ZNF750 overexpression combined with FGF14 knockdown groups two weeks after tumor cell implantation in mice. **i**, **j** Quantitative RT-PCR detection of *ZNF750* and *FGF14* expression in xenograft tumor cells. *p < 0.05, **p < 0.01, ***p < 0.001

To further confirm whether FGF14 functions as direct downstream of ZNF750, we mutated the binding site of ZNF750 in the promoter of *FGF14* (43 base pair upstream of transcription start site) (Fig. 6a). A luciferase reporter assay was performed, which showed that ZNF750 directly regulated the expression of *FGF14* in NPC cells (Fig. 6b). We then asked whether inhibiting FGF14 would reverse the tumor repressor function of ZNF750 in vitro and in vivo. After overexpressing *ZNF750* and blocking *FGF14* expression simultaneously in NPC cells, we found that *FGF14* knockdown promoted the growth of NPC cells that overexpressed ZNF750 (Fig. 6c–f). Moreover, the inhibition of NPC tumor growth in mice after *ZNF750* overexpression was reversed by *FGF14* knockdown (Fig. 6g–j). These data confirmed that FGF14 functions directly downstream of ZNF750 in NPC.

### The ZNF750-FGF14 signaling axis accelerates NPC cell apoptosis

**Fig. 7:**
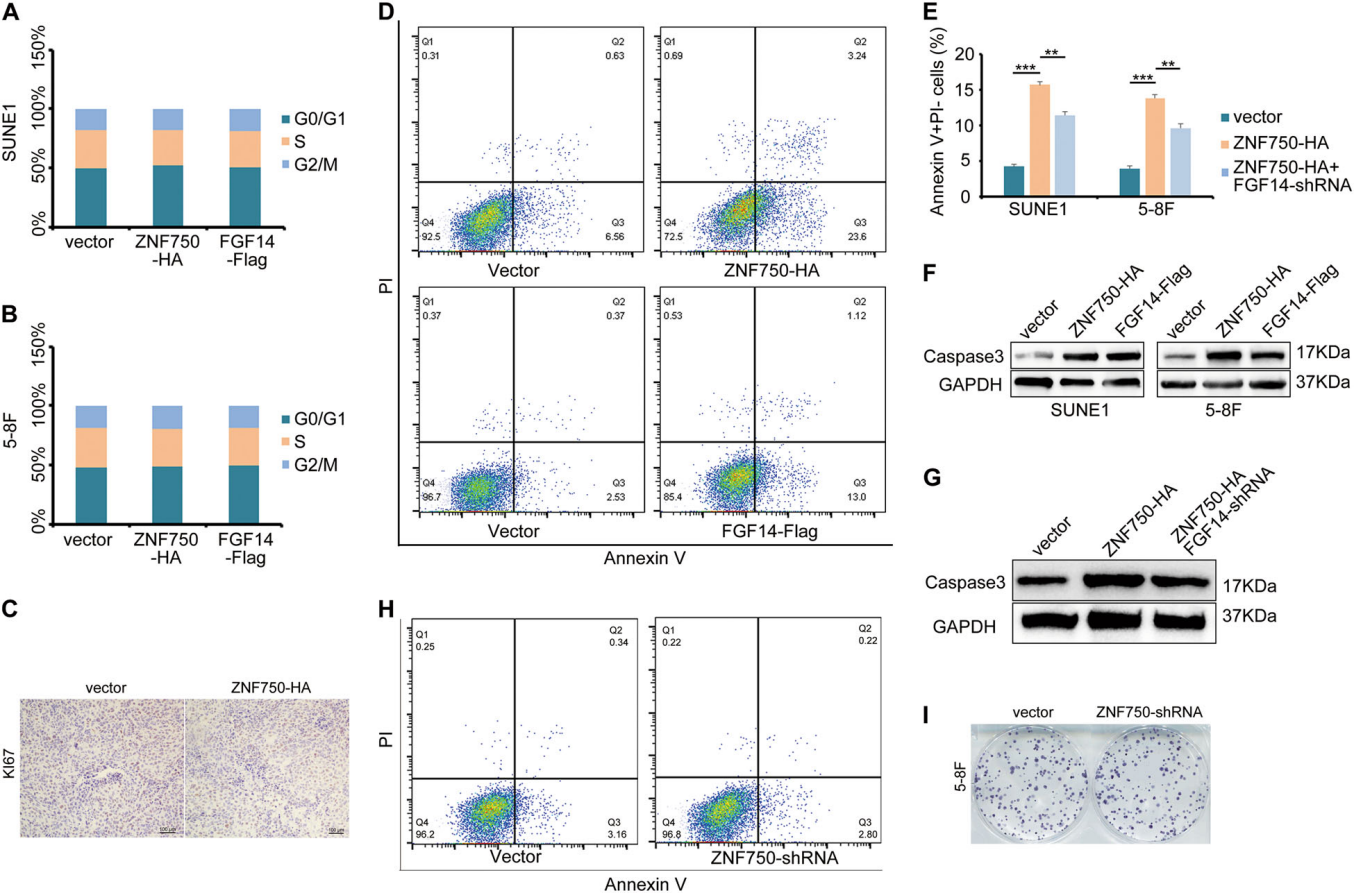
The ZNF750-FGF14 signaling axis promotes NPC cell apoptosis. **a**, **b** The cell cycle status of SUNE1 and 5–8 F cells after EdU staining. **c** Immunohistochemical staining of Ki67 in xenograft tumor cells. **d** Flow cytometry analysis of Annexin V and PI staining in SUNE1 cells with ZNF750 or FGF14 overexpression. **e** Quantification of the apoptotic cells in SUNE1 and 5–8 F cells. **f** The expression of cleaved Caspasd-3 in NPC cells with ZNF750 or FGF14 overexpression, as assessed by western blot. **g** Western blotting detection of cleaved Caspase-3 in xenograft tumor cells. **h** Flow cytometry examination of Annexin5 and PI staining in 5–8 F cells with ZNF750 knock down. **i** Colony formation assay of 5–8 F cells with ZNF750 knock down. **p < 0.01, ***p < 0.001

We next asked how the ZNF750-FGF14 signaling axis inhibited NPC cell growth. We firstly examined the cell cycle status of NPC cells with *ZNF750* or *FGF14* overexpression after 5-ethynyl-2′-deoxyuridine (EdU) incorporation, which showed that ZNF750 or FGF14 did not alter the cell cycle status (Fig. 7a, b). In xenograft tumors, Ki67 staining did not change significantly after *ZNF750* overexpression (Fig. 7c), indicating the proliferation capacity was unaffected. Then we examined whether cell apoptosis accounted for the impaired cell growth upon *ZNF750* or *FGF14* overexpression. Annexin V and PI staining revealed that both ZNF750 and FGF14 promoted NPC cell apoptosis (Fig. 7d, e). Moreover, knocking down FGF14 partially decreased the proportion of apoptotic cells, suggesting that ZNF750’s regulation of NPC cell apoptosis was dependent on FGF14 expression. Furthermore, the expression of cleaved Caspase-3 was also elevated in NPC cell lines and xenograft tumors (Fig. 7f, g), confirming that the ZNF750-FGF14 axis repressed NPC growth through promoting cell apoptosis. However, we also found that ZNF750 knock down did not influence on cell apoptosis or cell growth apparently (Fig. 7h, i). Considering the low expression level of ZNF750 in NPC, we speculated that the gene did not function in normal NPC cells.

**Fig. 8:**
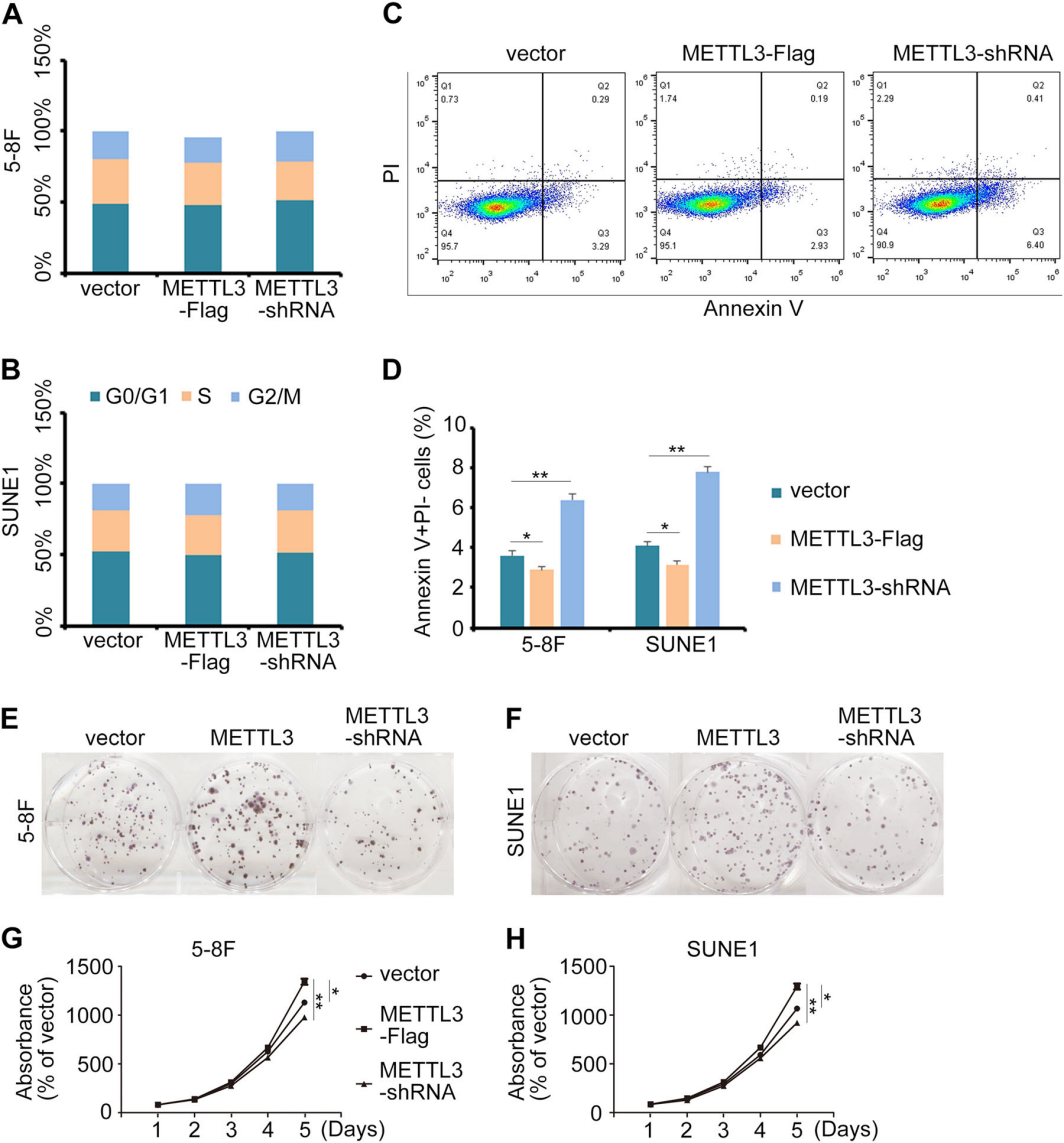
METTL3 influences NPC progression by inhibiting cell apoptosis. **a**, **b** The cell cycle status of SUNE1 and 5–8 F cells after EdU staining. **c** Flow cytometry analysis of apoptosis in 5–8 F cells with METTL3 ectopic expression or knockdown. **d** Quantification of apoptotic cells in the cytometry results. **e**, **f** Colony formation assay of NPC cells with METTL3 overexpression or knockdown. **g**, **h** CCK-8 assay of NPC cells with METTL3 overexpression or knockdown. *p *<* 0.05, **p *<* 0.01

METTL3 modulated *ZNF750* expression epitranscriptionally and served as a prognostic factor in HNSC; therefore, we tested whether METTL3 was involved in NPC progression. Similar to ZNF750, cell cycle status did not change upon manipulating *METTL3* expression (Fig. 8a, b), while cell apoptosis was significantly activated when *METTL3* was knocked down, and vice versa (Fig. 8c, d). Concomitantly, colony formation assay and cell growth analysis also illustrated that METTL3 functioned as an oncogene in NPC (Fig. 8e–h).

## Discussion

Until now, little was known about the effect of m^6^A in NPC. DNA methylation in NPC has been examined in previous studies^5,22–24^. As an important supplement to the central dogma, m^6^A-mediated RNA methylation and its role in cancer is only just beginning to be studied. Our work revealed that METTL3 mediated m^6^A modification blocked ZNF750 expression and thus promoted NPC growth, which presented an example showing m^6^A played essential regulatory roles post-transcriptionally in NPC. Considering that *ZNF750* is hypomethylated at its promoter and hypermethylated as mRNA, we hypothesize that epigenetic and epitranscriptional modifications may function together to modulate gene expression in some cases, complicating the study of the molecular mechanism governing NPC progression. Moreover, we identified *METTL3* as an oncogene in NPC. In addition to ZNF750, we cannot exclude the involvement of other genes in NPC progression whose expression levels are affected by METTL3-mediated m^6^A modification. Therefore, similar to studies on DNA methylation, more research is required to reveal the whole m^6^A modification landscape in NPC.

ZNF750 was reported to be indispensable for terminal epidermal differentiation through regulating KLF4 expression^25^. A previous study also revealed that ZNF750 acted as a tumor repressor capable of promoting apoptosis through large-scale genomic analysis in 4742 samples spanning 21 tumor types^17^. However, the functional mechanism of ZNF750 in cancer remains obscure. Our findings identified *FGF14* as the direct target gene of ZNF750, and the ZNF750-FGF14 signaling axis inhibited NPC growth through promoting cell apoptosis, which broadened our understanding of ZNF750 in repressing tumor development.

FGF/FGFR signaling is involved in a variety of cellular processes, including tumorigenesis^26,27^. FGF14, as a member of the non-secreted type FGF family, does not function as an FGF ligand^28^. A previous study revealed that FGF14 regulates spinocerebellar development, the loss of which leads to spinocerebellar ataxia in mice^29^. However, there are no reports on FGF14 in cancer. Our findings revealed that FGF14 was repressed in NPC patients, and served as the direct target of ZNF750. Moreover, blocking cell apoptosis is one of the main characteristics of cancer. Targeting and promoting apoptotic processes is regarded as an effective method for anti-cancer therapy^30–32^. Our study demonstrated that the ZNF750-FGF14 signaling axis promoted tumor cell apoptosis, which may help to identify new therapeutic targets in NPC. However, the functional mechanism of FGF14 still needs to be explored in future studies.

## Conclusion

The m^6^A modification in ZNF750 facilitates its downregulation in NPC, which promotes NPC progression. Overexpression of *ZNF750* blocked tumor cell growth in vitro and in vivo, while FGF14 functions as the downstream target of ZNF750 to regulate NPC apoptosis. These findings provide new insights into the molecular regulatory mechanism governing NPC progression, which may lead to new clinical treatment strategies in NPC.

## Materials and methods

### Ethical approval

This study was performed in accordance with ethical standards, according to the Declaration of Helsinki, and according to national and international guidelines. The study was approved by the ethics committee of Sun Yat-sen university Cancer center, and the approval number is YB2018-28.

### Patients and tumor tissue samples

Tumor samples were obtained from patients with pathologically confirmed NPC (*n* = 25) at Sun Yat-sen University Cancer Center. No patients received clinical treatment before sampling, and all patients provided the written informed consent.

### Cell lines

NP69, a human immortalized nasopharyngeal epithelial (NPEC) cell line, was cultured in keratinocyte serum-free medium (Invitrogen, Life Technologies, Grand Island, NY) with bovine pituitary extract (BD, Biosciences, USA). NPC cell lines 5–8 F, 6–10B, C666-1, HNE1, HONE1, S18, S26, and SUNE1 were cultured in RPMI-1640 (Invitrogen) supplemented with 5% FBS (Gibco, Carlsbad, CA, USA). The cells were seeded in 6-well plates the day before transfection, which was performed using Lipofectamine 3000 (Invitrogen), and the cells were harvested two days later.

### RNA extraction and reverse transcription-PCR (RT-PCR)

Total RNA from cell lines was extracted using the TRIzol reagent (Invitrogen). For the sorted cells from NPC biopsy samples, RNA was extracted using an RNeasy Micro kit (Qiagen, Hilden, Germany) following the manufacturer’s instructions. cDNA was synthesized using M-MLV reverse transcriptase (Promega, Madison, WI, USA), and amplified using SYBR Green qRT-PCR SuperMix-UDG reagents (Invitrogen) and a CFX96 instrument (Bio-Rad, Hercules, CA, USA). The genes were amplified using the following primers: *GAPDH* forward, 5′-GAAGGTGAAGGTCGGAGT-3′, and reverse 5′-GAAGATGGTGATGGGATTTC-3′, *ZNF750* forward, 5′-TACAGCCCCAGGAACATC-3′, and reverse 5′-GCTCCTTGCTGGGATTTT-3′, *FGF14* forward, 5′-TATGAAAGGGAACAGAGTAAAG-3′, and reverse 5′-CAGGACGAATAAGTCACAAC-3′, *METTL3* forward, 5′-AGGGTCTGGATTGTGATG-3′, and reverse 5′-CTGGGTCTAGTAGGTGGA-3′.

### ChIP-Seq

About 1 × 10^7^ SUNE1 cells with ZNF750-HA overexpression were harvested and treated according to the instructions of Pierce™ Magnetic ChIP kit (Thermo Fisher Scientific, Waltham, MA, USA). The cell extracts were collected and sent for sequencing by the Beijing Genomics Institute (BGI, Shenzhen, China). The qRT-PCR primers used for detecting *FGF14* in the ChIP assay were listed as follows: *FGF14* forward, 5′-CCACCCATCCTCATAGCG-3′, and reverse 5′-AGCATGAAGCCAAACAGA-3′.

### Western blot and immunofluorescence

Total protein were extracted using RIPA lysis buffer (Beyotime, China). Proteins were separated by SDS-polyacrylamide gel electrophoresis (SDS-PAGE), and transferred onto PVDF membranes (Millipore, Billerica, MA, USA). The membranes were then incubated with primary antibodies against the HA tag (1:2000, H6908, Sigma-Aldrich, Munich, Germany), Flag tag (1:2000, F2555, Sigma), METTL3 (1:1000, ab195352, Abcam, Cambridge, MA, USA), or GAPDH (1:1000, ab8245, Abcam) at 4 °C overnight. After incubation with species-matched secondary antibodies, the immunoreactive proteins were detected using chemiluminescence in a Gel imaging system (Bio-Rad, ChemiDoc MP Imaging System). For immunofluorescence, cells overexpressing *ZNF750* were seeded and cultured on cover glass, and then fixed by methanol after 24 h of culture. The cells were then reacted with anti-HA antibody (1:500, H6908, Sigma), followed with the corresponding secondary fluorescent antibody. The cells were viewed using confocal microscopy (Olympus, Tokyo, Japan).

### Stable cell line establishment and shRNA treatment

The *ZNF750*, *FGF14*, and *METTL3* coding sequences were cloned into the pSin-EF2-puro vector, separately. The stably overexpressing cell lines were obtained by puromycin screening and confirmed by western blotting. Short hairpin RNA (shRNA) targeting *FGF14* or *METTL3* were designed using an online tool (GPP Web Portal), and cloned into vector pLKO. The shRNA target sequences of the *ZNF750*, *FGF14*, and *METTL3* were listed below: ZNF750 target sequence, 5′-GAGTTCCCAAGTGCCCTAAAT-3′, FGF14 target sequence, 5′-ACCAGGTTATATTGCAGGCAA-3′, METTL3 target sequence, 5′-GCCAAGGAACAATCCATTGTT-3′.

### Cell proliferation, colony formation assay, and cell invasion

The CCK-8 assay was used to detect cell proliferative ability. 1 × 10^3^ cells were seeded into 96-well plates, incubated for 0–4 days, and stained using the CCK-8 kit (Dojindo, Tokyo, Japan). The absorbance values were determined at 450 nm using a spectrophotometer. For the colony formation assay, about 300 cells were seeded into 6-well plates. After 7–10 days of culture, the cells were fixed by methanol and stained with crystal violet. For the cell invasion assay, 3 × 10^3^ cells were seeded into the 24-well Transwell chambers (Corning, NY, USA). The medium was supplemented with 10% FBS and placed in the lower chambers. After 14–18 h of culture, the chambers were collected and the cells on lower surface of the chambers were fixed by methanol and stained with crystal violet for observation.

### EdU staining and cell apoptosis detection

For EdU staining, 1 × 10^5^ cells were seeded into 6-well plates. After the cell density reached about 90%, EdU (10 μM, C0088S, Beyotime) was added, and the staining procedure was operated following the manufacturer’s instructions. The cell cycle status were analyzed by flow cytometry (CytoFLEX 1, Beckman Coulter, Brea, CA, USA). For cell apoptosis analysis, 1 × 10^5^ cells were seeded into 6-well plates. Before the cell density reached about 90%, the cells were collected and stained with Annexin V and propidium iodide (PI) (C1062, Beyotime), and then analyzed by flow cytometry (CytoFLEX 1).

### Luciferase reporter assay

The wild-type and mutated *FGF14* promoter (1000 base pairs upstream of transcription start site) were cloned into firefly luciferase-expressing vector psiCHECK™ (Promega). For the luciferase reporter assay, cells were co-transfected with *ZNF750* and FGF14 wild-type or mutated promoter reporter vectors. The luciferase activity was examined by a Dual-luciferase Reporter System (Promega) following the manufacturer’s instructions.

### RNA immunoprecipitation (RIP)

About 1 × 10^7^ NPC cells with *METTL3* knocked-down or overexpressed were harvested and manipulated as the instructions of the Magna RIP™ kit (Millipore). Anti-m^6^A antibody (10 μg antibody for 5 μg of mRNA, Synaptic Systems, 202003) was used, and the immunoprecipitated RNA extracts were reverse-transcribed and examined by qRT-PCR. The primers for *ZNF750* used in m^6^A-RIP-qRT-PCR were listed: forward 5′-TCCAGCAATATCCCTCTAACC-3′, and reverse 5′-CCTCAGGAATTGGACTTTCG-3′.

### Animal experiments

BALB/c-nu mice (4–6 weeks old, female) were purchased from Charles River Laboratories (Beijing, China), and SUNE1-vector-luciferase or SUNE1-ZNF750-luciferase cells (1 × 10^6^) were subcutaneously injected into the dorsal or ventral flank. Tumor cells were monitored 7–10 days later. Luciferin was diluted into 15 mg/ml using phosphate-buffered saline (PBS), and 100 μl of the solution was intraperitoneally injected into each mouse. Five minutes later, the mice were anesthetized and observed using an animal imaging system (IVIS Lumina LT, PerkinElmer, Waltham, MA, USA). All animal research was performed in accordance with the detailed rules approved by the Animal Care and Use Ethnic Committee of Sun Yat-sen University Cancer Center and all efforts were made to minimize animal suffering.

### Immunohistochemistry

For immunohistochemistry, xenograft tumors were fixed with paraformaldehyde and embedded in paraffin. The samples were sectioned and mounted on slides. The sectioned slides were incubated by anti-KI67 antibodies (1:500, ab15580, Abcam) at 4 °C overnight. Then the sections were incubated with biotinylated secondary antibody bound to a horseradishperoxidase complex. The antibody was visualized by adding 3,3-diaminobenzidine, and the sections were counterstained with hematoxylin.

### Statistical analysis

Statistical analyses were performed using SPSS 17.0 (SPSS Inc., Chicago, IL, USA). All data shown are representative of at least three independent experiments, and values are expressed as the mean ± SD. Differences between two groups were analyzed using the two-tailed unpaired Student’s *t*-test; *p* < 0.05 was considered significant. For the correlation analysis, Pearson analysis method was used. All data in our study have been recorded at Sun Yat-sen University Cancer Center for future reference (RDDB2018000415).

## Electronic supplementary material

Supplementary figure 1

Supplementary Table 1

Supplementary Table 2

Supplementary figure legends
