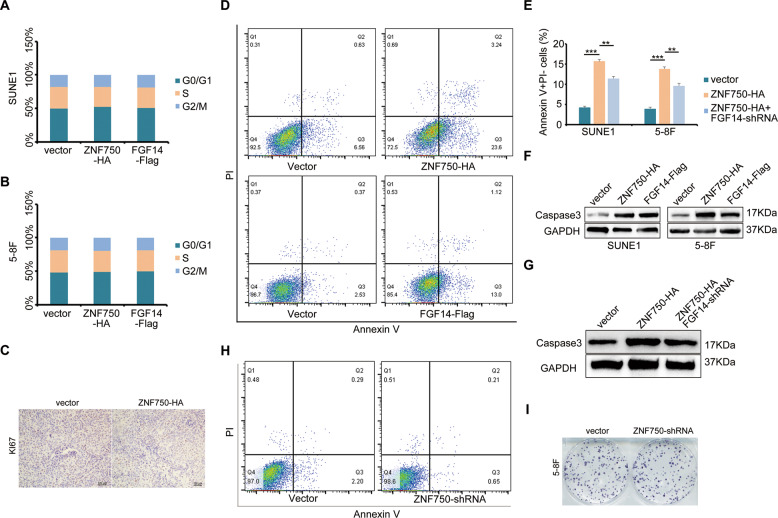# Correction: m^6^A-mediated ZNF750 repression facilitates nasopharyngeal carcinoma progression

**DOI:** 10.1038/s41419-022-04544-y

**Published:** 2022-01-26

**Authors:** Panpan Zhang, Qiuping He, Yuan Lei, Yingqin Li, Xin Wen, Mengzhi Hong, Jian Zhang, Xianyue Ren, Yaqin Wang, Xiaojing Yang, Qingmei He, Jun Ma, Na Liu

**Affiliations:** 1 State Key Laboratory of Oncology in South China, Collaborative Innovation Centre of Cancer Medicine, Guangdong Key Laboratory of Nasopharyngeal Carcinoma Diagnosis and Therapy, Sun Yat-sen University Cancer Center, 510060 Guangzhou, China; 2 Zhongshan School of Medicine, Sun Yat-sen University, 510080 Guangzhou, China; 3 Guangdong Provincial Key Laboratory of Stomatology, Guanghua School of Stomatology, Sun Yat-sen University, 510055 Guangzhou, Guangdong China

Correction to: *Cell Death Dis* (2018); 9:1169 10.1038/s41419-018-1224-3, published online 05 December 2018

The original version of this article unfortunately contained a mistake in Fig. 7. The authors apologize for the mistake. The correct figure can be found below.