# Supplementary figures and images for: m6A-mediated ZNF750 repression facilitates nasopharyngeal carcinoma progression
Source: Cell Death Dis. 2018 Dec 5;9(12):1169. doi: 10.1038/s41419-018-1224-3 (PMC6281568; doi:10.1038/s41419-018-1224-3)

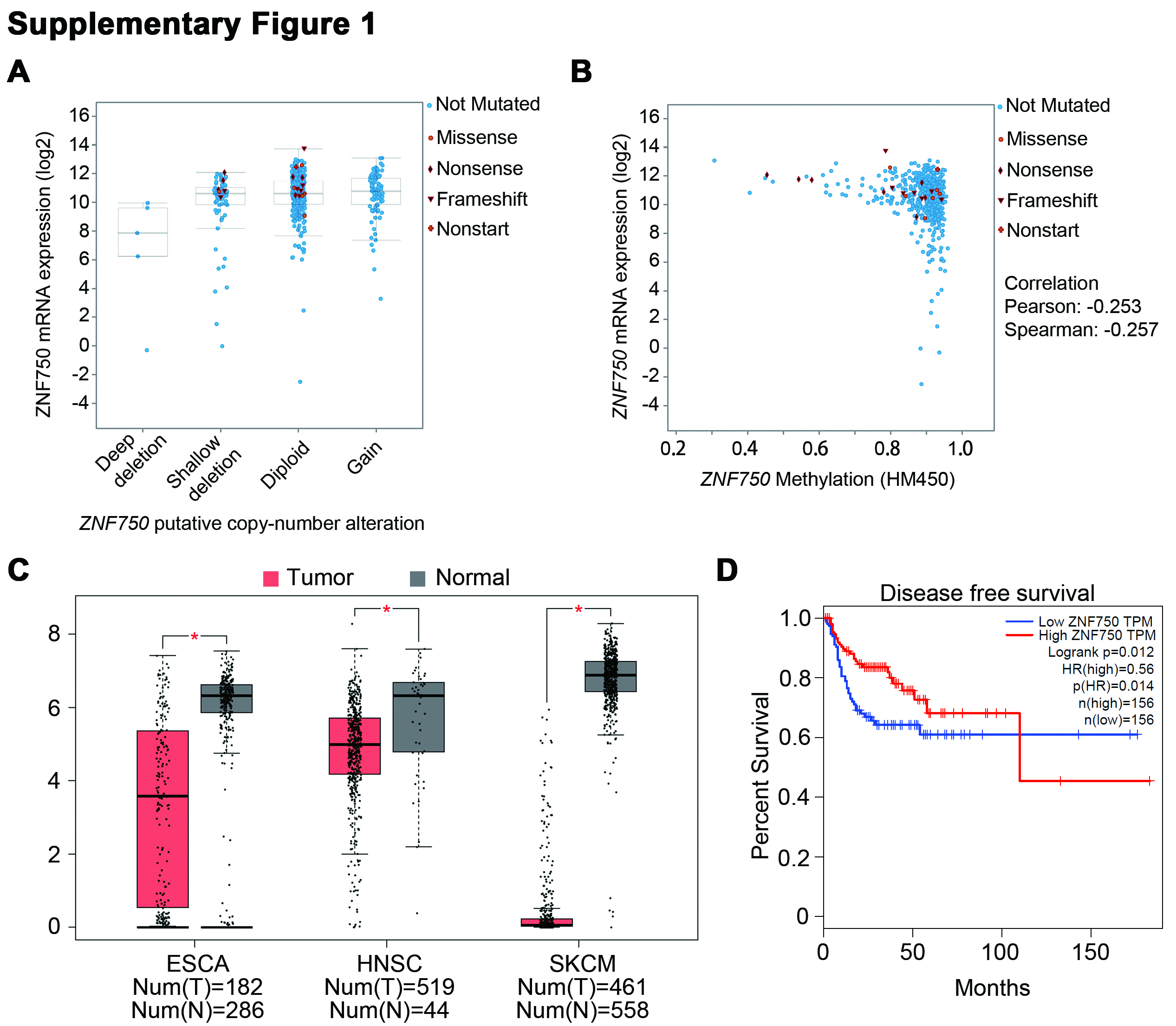

Supplement: Supplementary file 1 — Supplementary figure 1 [file 41419_2018_1224_MOESM1_ESM.jpg]
