# Supplementary material for: m6A-mediated ZNF750 repression facilitates nasopharyngeal carcinoma progression
Source: Cell Death Dis. 2018 Dec 5;9(12):1169. doi: 10.1038/s41419-018-1224-3 (PMC6281568; doi:10.1038/s41419-018-1224-3)
Supplement: Supplementary file 4 — Supplementary figure legends [file 41419_2018_1224_MOESM4_ESM.docx]

**Supplementary Figure 1. ZNF750 serves as a tumor repressor and correlates with better disease-free survival in HNSC.** (A, B) *ZNF750* mRNA expression and copy-number alteration or methylation status in HNSC in the cBioPortal dataset. (C) The mRNA expression of *ZNF750* in the TCGA dataset. (D) Disease-free survival (DFS) analysis in HNSC according to the ZNF750 expression in the TCGA dataset, ** p*<0.05.

**Table 1. The list of hypomethylated genes in patients with NPC (GSE52068)**. Compared with normal people healthy controls, the hypomethylated genes in NPC patients with an absolute value >2 were screened.

**Table 2. The downstream targets of ZNF750 as revealed by ChIP-Seq.**
